# Supplementary material for: Analysis of PPARγ Signaling Activity in Psoriasis
Source: Int J Mol Sci. 2021 Aug 10;22(16):8603. doi: 10.3390/ijms22168603 (PMC8395241; doi:10.3390/ijms22168603)
Supplement: Supplementary file 1 [file ijms-22-08603-s001.zip › Supplemental materials_Analysis of PPARg signaling activity in psoriasis/Pathway models/Models images and html files/PPARG expressed in cells assotiated with psoriasis/225457.html]

epithelial cell


# CellType epithelial cell

|  |  |
| --- | --- |
| URN | urn:agi-ncimcelltype:C0014597 |
| Total Entities | 50 |
| Connectivity | 11193 |
| Name | epithelial cell |

---

|  |  |
| --- | --- |
| ChildConcepts | mammary epithelial cell |
|  | ciliated cell |
|  | epidermis cell |
|  | ameloblast |
|  | columnar cell |
|  | spongioblast |
|  | pneumocyte |
|  | pericyte |
|  | corneal epithelial cell |
|  | ovarian epithelial cell |
|  | Hensen cell |
|  | endothelial cell |
|  | cholangiocyte |
|  | kidney tubule cell |
|  | type 2 epithelial cell |
|  | type 1 epithelial cell |
|  | pulmonary ionocyte |
|  | airway epithelial basal cell |
|  | buccal cell |
|  | podocyte |
|  | squamous cell |
|  | basaloid cell |
|  | secretory cell |
|  | epithelioreticular cell |
|  | Claudius cell |
|  | cuboidal cell |
|  | sebocyte |
|  | myoepithelial cell |
|  | olfactory supporting cell |
|  | renal tubular epithelial cell |
|  | reserve cell |
|  | thymic epithelial cell |
|  | thymic medullary epithelial cell |
|  | transitional epithelial cell |
|  | meningothelial cell |
|  | mesothelial cell |
|  | ependyma cell |
|  | epithelial cell rests of Malassez |
|  | sustentacular cell |
|  | amnion cell |
|  | cornea cell |
|  | crypt cell |
|  | endometrium cell |
|  | epithelium basal cell |
|  | intestine epithelium cell |
|  | pigment cell |
|  | glomerulus epithelium cell |
|  | lens epithelium cell |
|  | mouth epithelium cell |
|  | prostate epithelium cell |

---

|  |  |
| --- | --- |
| Pathway | Elevated Receptors -> Expression Targets in Epididymis |
|  | PPARG expressed in cells assotiated with psoriasis |
|  | Neighbors of epithelial cell |
|  | 4\_2\_Airway smooth muscle cells proliferation and airway tissue remodeling |

---

|  |  |
| --- | --- |
| MedScan ID | 10000165 |

---

|  |  |
| --- | --- |
| Alias | duct cells |
|  | epithelium cell |
|  | ductal epithelial cell |
|  | ductal epithelial cells |
|  | ductal cell |
|  | duct cell |
|  | epithelial cells |
|  | epithelial cell |
|  | ductal cells |
|  | epithelium cells |

---

|  |  |
| --- | --- |
| NCIm ID | C0014597 |

---
